# Emerging Insights into Granulomatous and Amyloidogenic Cardiomyopathies

**DOI:** 10.3390/jcm14124208

**Published:** 2025-06-13

**Authors:** Syed Bukhari, Adnan Younus, Zubair Bashir

**Affiliations:** 1 Department of Medicine, Division of Cardiology, Johns Hopkins University School of Medicine, Baltimore, MD 21287, USA; 2 TidalHealth Peninsula Regional, Salisbury, MD 21801, USA; adnan.younus@tidalhealth.org; 3 Division of Cardiology, University of Texas Medical Branch, Galveston, TX 77555, USA; shahid.zubair08@gmail.com

**Keywords:** cardiac amyloidosis, cardiac sarcoidosis, granulomatous cardiomyopathy, cardiac magnetic resonance, echocardiography, nuclear scintigraphy, PET

## Abstract

**Background:** Granulomatous and amyloidogenic cardiomyopathies are infiltrative conditions that can be fatal if left untreated. Among these, cardiac amyloidosis and cardiac sarcoidosis are significant but often underdiagnosed causes of heart failure, each serving as cardiac manifestations of broader systemic diseases. Advancements in imaging techniques and the emergence of novel therapies—particularly for cardiac amyloidosis—have brought these conditions into sharper focus for both clinicians and researchers. **Methods:** We conducted a comprehensive review of the literature by searching databases including PubMed and Scopus for studies published since 1990 regarding clinical features, diagnostic techniques, and treatment strategies for cardiac amyloidosis and cardiac sarcoidosis. Studies were selected based on relevance to imaging methods, including echocardiography, cardiac magnetic resonance imaging (CMR), positron emission tomography (PET), and technetium-labeled nuclear scintigraphy, as well as treatment modalities for both conditions. **Results:** Imaging techniques, particularly CMR, technetium-labeled nuclear scan, and PET, were found to be crucial for the early identification and differentiation of cardiac amyloidosis and cardiac sarcoidosis. Distinct late gadolinium enhancement patterns were observed in CMR along with morphological differences, aiding in diagnosis. Technetium-labeled nuclear scintigraphy can definitively distinguish between subtypes of cardiac amyloidosis in the absence of paraproteinemia. Early diagnosis has been shown to significantly improve patient outcomes. Early treatment can reduce morbidity in both cardiomyopathies. **Conclusions:** Multimodality imaging can help in the early detection of cardiac amyloidosis and cardiac sarcoidosis. Treatment strategies differ substantially: cardiac amyloidosis is primarily managed with disease-modifying therapies for the transthyretin subtype and chemotherapy/stem cell transplant for the AL subtype, while cardiac sarcoidosis is treated with corticosteroids and immunosuppressive drugs to reduce inflammation. Early and accurate diagnosis through advanced imaging techniques is critical to improving outcomes for patients with these conditions.

## 1. Introduction

Granulomatous and amyloidogenic cardiomyopathies are infiltrative conditions that can be fatal if left untreated. Among these, cardiac amyloidosis (CA) and cardiac sarcoidosis (CS) are significant but often underdiagnosed causes of heart failure with distinct pathogenetic mechanisms, each serving as cardiac manifestations of broader systemic diseases. The emergence of highly specialized imaging techniques has revealed a higher-than-expected prevalence of these conditions. While nuclear scintigraphy with bone-avid tracers can accurately differentiate between subtypes of CA, highly sophisticated tissue characterization with cardiac magnetic resonance (CMR) along with the detection of granulomatous inflammation through positron emission tomography (PET) has helped increasingly diagnose subclinical CS. In parallel, the development of novel therapies, particularly for CA, has heightened attention and research in these conditions. In this review, we will discuss the pathophysiology, clinical characteristics, diagnostic imaging, and therapies of both conditions.

### 1.1. Pathophysiology of CA

In CA, pathophysiology involves the synthesis of misfolded proteins which form amyloid fibrils that infiltrate the myocardium [1]. These fibrils accumulate in the extracellular matrix of the heart, disrupting normal myocardial architecture and function. Amyloid deposition leads to stiffening of the myocardium, impairing both diastolic and systolic function, which contributes to heart failure and arrythmias [2]. In >90% of cases, amyloidogenesis involves two proteins, transthyretin (ATTR) and immunoglobulin light chain (AL).

TTR is a tetrameric protein primarily produced in the liver that plays a role in transporting thyroxine and vitamin A. In ATTR, genetic mutations (ATTRv) or aging-related changes (ATTRwt) lead to instability in the TTR tetramer, causing dissociation into monomers that subsequently misfold and aggregate into amyloid fibrils [3]. ATTRwt commonly presents as heart failure with preserved ejection fraction (HFpEF), with patients experiencing symptoms such as dyspnea, fatigue, and exercise intolerance [4]. Other notable symptoms include peripheral edema, orthopnea, and paroxysmal nocturnal dyspnea. As many as ~1/3 of patients hospitalized due to HFpEF are found to have ATTRwt [5]. In addition to heart failure, patients may experience arrhythmias, especially atrial fibrillation, and conduction disturbances, such as bundle branch blocks or atrioventricular (AV) block (AVB), due to the amyloid infiltration of the cardiac conduction system [6]. One of the distinguishing features of ATTRwt is its insidious onset, often without clear preceding risk factors for heart failure. It is also more common in men, with elderly Caucasians being particularly affected. Although ATTRwt primarily affects the heart, it can also involve other organs, albeit less frequently than the familial forms of amyloidosis.

ATTRv is a genetically heterogeneous condition, characterized by a wide range of clinical manifestations, depending on the specific mutation, as well as the affected organ systems. The disease often presents as either cardiomyopathy or neuropathy, or a combination of both. The V122I mutation is the common mutation in the United States, which behaves like ATTRwt, affecting predominantly elderly men [7]. The key phenotypic difference is that, unlike ATTRwt which affects predominantly Caucasians, the V122I mutation almost exclusively affects individuals of African American race [7,8]. The most common mutation worldwide, V30M, is caused by a methionine for valine substitution at residue 30 of the mature TTR protein, which is encoded by the *TTR* gene located on chromosome18q12.1, and could manifest as neuropathy and/or cardiomyopathy [9]. It presents either as early-onset or late-onset disease, and while the worldwide prevalence is unknown, data are available for countries like Portugal, Japan, and Sweden where the disease is endemic [9]. T60A remains the predominant genetic mutation identified in Irish patients, who typically present in the seventh decade with an already manifest neuropathy phenotype, largely predating their cardiac phenotype, which is dominated by heart failure [10].

AL is a hematologic disorder characterized by the deposition of misfolded immunoglobulin light chains as amyloid fibrils in various organs, including the kidneys, heart, liver, and nervous system. The pathogenesis of AL begins with the clonal expansion of plasma cells in the bone marrow, which produce abnormal light chains [11]. These light chains are produced in excess or in an improperly folded form and are secreted into the bloodstream. Once in circulation, the light chains can undergo misfolding, leading to the formation of amyloid fibrils. The heart is the second most commonly affected organ after the kidneys, and is the key determinant of prognosis. AL is a more aggressive disease than ATTR, with a 6-month survival from the onset of cardiac symptoms if untreated [12].

### 1.2. Pathophysiology of CS

CS, a prototype of granulomatous cardiomyopathy, is a rare manifestation of sarcoidosis, characterized by the infiltration of the myocardium by noncaseating granulomas. The exact cause of CS is not fully understood but is thought to result from a complex interplay of genetic, environmental, and immunologic factors [13,14]. It is commonly associated with systemic sarcoidosis, particularly pulmonary involvement, but isolated cardiac involvement can also occur. While 5% of patients with CS are symptomatic, up to 1/3 have a silent disease that is detected after imaging or autopsy [15]. In CS, granulomatous inflammation typically affects the interventricular septum, left ventricle (LV), and occasionally the right ventricle, leading to a variety of clinical manifestations. Arrhythmias and conduction abnormalities are more common, while heart failure and sudden cardiac death (SCD) are less common. The extent and location of myocardial involvement influence the severity of symptoms; patients with limited myocardial involvement may remain asymptomatic, while those with more widespread disease are at an increased risk of significant conduction disturbances and heart failure [16]. The condition predominantly affects young adults, with a higher prevalence in individuals of African American and Northern European descent.

## 2. Clinical Characteristics in CA

Early diagnostic workup for CA relies heavily on clinical evaluation, including a comprehensive history and EKG. A patient’s history can provide valuable clues that help differentiate it from other heart conditions and guide further investigations. Extracardiac manifestations can particularly be useful, including carpal tunnel syndrome, spinal stenosis, hip or knee replacement, prior shoulder surgery, proteinuria, and peripheral or autonomic neuropathy causing orthostatic hypotension (Table 1). Carpal tunnel syndrome is one of the earliest features of CA and precedes cardiac involvement by ~5–10 years [17]. Low-voltage EKG, although not pathognomonic, can be seen, which is a result of the poorly conducting amyloid fibrils infiltrating the myocardium [18]. Importantly, low voltage is detected only in ~35% of ATTR and 55% of AL patients, and therefore the absence of low voltage criteria does not rule out CA [19]. It often manifests in advanced disease stage and carries prognostic implications [19]. Low-voltage EKG can become more significant in the presence of LV ‘hypertrophy’, as this discrepancy is a peculiar feature seen in CA and strengthens suspicion for CA. Other EKG features that are reflective of the generalized infiltrative nature of this disease include atrial fibrillation, pseudo-infarct pattern, and first-degree AV block [20,21]. Atrial fibrillation is the most common arrhythmia in CA, and this high prevalence could be attributed to atrial enlargement due to high filling pressures and atrial myopathy secondary to direct amyloid infiltration [22,23]. Ventricular tachyarrhythmias (VT) and bradyarrhythmias are also seen [24,25]. On laboratory evaluation, the persistent elevation of high-sensitivity troponin due to myocardial injury is almost invariably seen in CA and has prognostic implications [26]. Several mechanisms have been proposed in myocardial injury in CA, including amyloid-related causes such as amyloid precursor toxicity, amyloid interstitial infiltration, and amyloid vascular involvement, as well as non-amyloid-related causes such as diastolic and systolic dysfunction, heart failure, atherosclerotic coronary artery disease, and other supply–demand imbalances such as tachyarrhythmias and hypotension [26].

## 3. Clinical Characteristics in CS

The presence of extracardiac sarcoidosis, particularly in the lungs, raises suspicion for CS in the presence of any cardiac symptoms. In patients with CS, various types of arrhythmias have been reported, among which AVB is the most common while VT/ventricular fibrillation (VF) is the second most common initial presentation [27]. AVB results from infiltration of the interventricular septum due to sarcoid granuloma or scar tissue. It has been reported that SCD due to VT/VF or AVB and related complications accounts for 30–65% of deaths in sarcoidosis [28]. The risk of SCD is significant in CS presenting with high-grade AVB with or without VT or LV dysfunction, and the risk for SCD is high in these patients during a 5-year follow-up [29]. Therefore, detecting cardiac involvement and an early diagnosis with treatment is one of the most important issues, especially because immunosuppressive treatment substantially enhances prognosis in such patients. As sarcoidosis is typically a disease of middle-aged persons and predominantly women, the clinical suspicion of CS should be high in middle-aged women with “idiopathic” higher degree AVB.

## 4. Echocardiographic Features in CA

Echocardiography is a valuable and reproducible imaging modality for identifying the structural and functional abnormalities associated with CA [30]. This infiltrative disease typically manifests as myocardial thickening with restrictive physiology and elevated filling pressures, in the absence of significant aortic stenosis or uncontrolled hypertension [30]. Characteristic findings include a septal wall thickness exceeding 12 mm and a speckled appearance of the myocardium due to amyloid deposits [31,32]. Tissue Doppler imaging often reveals a restrictive filling pattern, with markedly reduced myocardial velocities (e’, a’, and s’ velocities typically <5 cm/s) [30]. The presence of pericardial effusion further supports the diagnosis [30].

Biatrial enlargement is commonly observed, reflecting increased filling pressures, and all phases of left atrial strain (reservoir, conduit, and contraction phases) are significantly reduced [33,34]. Interatrial septal thickening exceeding 6 mm has been reported to have 100% specificity for diagnosing CA [32], providing a strong diagnostic clue. In addition, unexplained right ventricular free wall thickening and chamber dilatation raise suspicion for CA [32]. Speckled tracking echocardiography often reveals marked impairment in global longitudinal strain (GLS) of the left and right ventricles, particularly at the basal and mid-segments, with relative apical sparing. This distinctive pattern produces the “cherry-on-top” appearance on the GLS bull’s-eye plot, a hallmark feature of CA [35,36].

## 5. Echocardiographic Features in CS

Two-dimensional echocardiography is often the initial screening tool for CS. In a classic clinical scenario, echocardiography is typically prompted by cardiac symptoms (palpitations, chest pain, presyncope, and syncope) and/or abnormal EKG in an individual with preexisting extra-cardiac sarcoidosis [37]. The echocardiographic features in CS are often nonspecific. Any echocardiographic abnormality, when considered in the context of presence of extracardiac sarcoidosis, should raise suspicion for CS and warrant further investigation with more advanced imaging modalities. These echocardiographic abnormalities may include LV chamber dilation, mild LV wall thickening or even LV thinning in some cases, diastolic dysfunction, trivial pericardial effusions, and LV aneurysm. LV systolic dysfunction is an important feature in CS, although usually observed late in the disease course [38]. The presence of wall motion abnormalities (WMAs) in a noncoronary distribution could serve as an important indicator of cardiac involvement in CS and could be present in up to 50% patients at the time of diagnosis of CS [39]. LV free wall and interventricular septum are most commonly involved. The most characteristic finding is thinning or scarring of the basal portion of the interventricular septum and associated akinesis, present in up to 4% of patients with a CS diagnosis [39,40].

Importantly, 2D echocardiography demonstrates poor sensitivity in detecting early, mild, or focal CS [41]. Myocardial deformation imaging has been proposed as a sensitive tool to detect myocardial dysfunction before a decrease in LV ejection fraction (LVEF) occurs [42]. The early impairment of LV GLS reflects the disruption of the myofibrils that are primarily organized longitudinally, with the greatest concentration in the subendocardial layer [43]. While CS preferentially affects the epicardial and mid-myocardial layers, patients with CS still demonstrate an early impairment of LV GLS.

Echocardiographic features also have prognostic implications in CS. Patients with reduced LVEF have poorer prognosis compared to those with preserved LVEF [44]. Impaired LV has been shown to have incremental prognostic value for predicting major adverse cardiac events (defined as death, VT, heart failure hospitalization, or transplantation) in patients with systemic sarcoidosis [45]. Finally, right ventricular involvement has been associated with higher risk of VA and death, and right ventricular free wall longitudinal strain has been recognized as an important surrogate of disease activity and prognosis [46].

## 6. Cardiac Magnetic Resonance in CA

Cardiac magnetic resonance (CMR) imaging is the gold standard noninvasive modality for diagnosing CA, offering exceptional spatial resolution and comprehensive myocardial tissue characterization [47,48]. Key features include late gadolinium enhancement (LGE), T1 and T2 mapping, and extracellular volume (ECV) quantification. LGE reflects gadolinium contrast accumulation in the myocardial interstitium, with delayed washout in amyloid-laden areas, often displaying a noncoronary artery territorial distribution. Patterns of LGE include subendocardial, diffuse, focal, and transmural enhancement, with transmural involvement indicating worse prognosis. The subendocardial pattern is particularly characteristic of CA, making LGE a cornerstone of its diagnosis [49,50].

T1 mapping allows the early detection of amyloid infiltration before overt myocardial thickening, with prolonged (pre-contrast) T1 relaxation times serving as a diagnostic and prognostic marker [51,52,53]. The pre- and post contrast T1 relaxation times measure the extent of extracellular matrix expansion, which helps differentiate CA from other fibrotic myocardial conditions and provides an objective metric for assessing disease burden and treatment response [54,55,56]. Complementing these techniques, T2 mapping quantifies myocardial edema, reflecting inflammation or active disease, and contributes additional prognostic information [57].

## 7. Cardiac Magnetic Resonance in CS

CMR is an excellent tool for the diagnosis and prognostication of patients with suspected CS, offering a multi-dimensional assessment of cardiac involvement that allows for a noninvasive detection of scar, biventricular function, edema, and myocardial perfusion defects. CMR offers the advantages of high spatial resolution, excellent soft-tissue contrast, and the use of non-ionizing radiation. While CMR can easily recognize morphological abnormalities such as areas of wall thinning or aneurysm, the foremost diagnostic technique to detect CS by CMR relies on identifying areas of mid-wall and subepicardial late gadolinium enhancement (LGE) [58]. Rarely, CS can also cause subendocardial LGE, thus mimicking an infarct pattern. Patchy LGE in a noninfarct pattern is a nonspecific finding, which is also seen in other diseases such as scars from previous myocarditis or from fibrosis in idiopathic cardiomyopathy, but this finding in the presence of preexisting extracardiac sarcoidosis would account for the presence of CS unless proven otherwise [58]. Features of LGE that typically favor the diagnosis of CS include multifocal involvement and the involvement of the basal anteroseptum and inferoseptum, demonstrating contiguous extension into the right ventricle [59]. A transmural pattern is significantly more common in patients with an LVEF of 35% or lower than in those with an LVEF exceeding 35% [60]. LGE also has prognostic value and is associated with future cardiovascular death and VT [61].

CMR has the potential to assess the inflammatory aspect of CS. By incorporating T2-weighted imaging and T2 mapping, CMR can detect edema and inflammation [62]. Although T2-weighted CMR has been suggested as a potential alternative to positron emission tomography (PET) for detecting inflammation and tracking therapeutic response, its lower signal-to-noise ratio presents a challenge, necessitating further clinical validation [62,63].

## 8. Nuclear Imaging with Bone-Avid Tracers

Nuclear scintigraphy with technetium-labeled tracers, including 99mTc-pyrophosphate (PYP), is a key imaging modality for diagnosing ATTR [64]. This technique has demonstrated high sensitivity and specificity when combined with serum immunofixation electrophoresis and serum free light chain testing to exclude monoclonal gammopathy [65]. The use of Tc-PYP scan has significantly reduced the need for histological confirmation of ATTR in most cases, although AL still requires histological confirmation [65].

The diagnostic utility of Tc-PYP scintigraphy relies on the affinity of ATTR deposits for the radioactive tracer, which is visualized by comparing tracer uptake in the myocardium with uptake in the rib cage (bone). Grade 2 uptake (myocardial uptake equal to bone) and Grade 3 uptake (myocardial uptake greater than bone) are highly specific for ATTR in the absence of paraproteinemia [66]. The heart-to-contralateral lung (H/CL) ratio adds one more layer to the accuracy of diagnosis, although there can be discrepancy between planar image and the H/CL ratio [67]. SPECT is always performed to confirm myocardial uptake and to distinguish it from blood pooling. This noninvasive imaging modality has become an essential tool in diagnosing and differentiating ATTR from other types of CA [68] (Table 2).

## 9. Positron Emission Tomography

Fluorine-18 fluorodeoxyglucose (FDG) PET has become an essential imaging technique for diagnosing and evaluating CS, especially in situations where standard methods like echocardiography or MRI may not provide clear results. FDG-PET is particularly useful in detecting areas of granulomatous inflammation in the tissues. In CS, the granulomas contain activated macrophages that exhibit increased glucose metabolism, which is effectively captured by FDG, allowing for the visualization of both cardiac and extracardiac disease involvement [69]. This imaging modality is highly sensitive in cases with advanced disease or active inflammation, offering valuable prognostic information regarding the extent and severity of myocardial involvement. Additionally, FDG-PET can detect myocardial inflammation that may remain undetected by conventional imaging, particularly in early or subclinical stages of the disease [70]. It is also a useful tool for differentiating between areas of active inflammation and scar tissue, which aids in monitoring treatment progress, especially during corticosteroid or immunosuppressive therapy [71]. However, while FDG-PET is a highly specific method for identifying active CS, its sensitivity can be affected by factors such as prior corticosteroid treatment, which may reduce metabolic activity, and the need for specific dietary preparation, including prolonged fasting, dietary modifications, and intravenous heparin to minimize normal myocardial glucose uptake. Despite these limitations, FDG-PET continues to be a vital tool for both diagnosing and managing CS, offering critical insights into disease activity and informing therapeutic decisions (Table 3).

## 10. Disease-Modifying Treatments

### 10.1. Therapies in ATTR

Therapies for ATTR that are FDA-approved are TTR silencers and TTR stabilizers. TTR silencers, such as RNA interference (RNAi) therapies, work by inhibiting TTR production through the degradation of TTR messenger RNA (mRNA) [75]. Patisiran, an RNAi agent, was the first of its kind to gain FDA approval for treating ATTR-related polyneuropathy. In a landmark trial comprising 225 patients who underwent randomization (148 to the patisiran group and 77 to the placebo group), patisiran treatment resulted in significant improvements in neuropathy, quality of life, walking, nutritional status, and activities of daily living. [76]. There was also evidence that patisiran improved cardiac manifestations of ATTRv, as indicated by echocardiographic measures of cardiac structure and function and a reduction in NT-proBNP levels [76]. The authors proposed that the increased gait speed in patients who received patisiran may have resulted from favorable effects on both the neuropathic and cardiac aspects of the disease.

Another TTR silencer, inotersen, is an antisense oligonucleotide that targets TTR mRNA, which has demonstrated the slowing of neuropathy progression in patients with ATTRv, including those with cardiac involvement [77]. The NEURO-TTR trial showed that weekly subcutaneous injections of inotersen (300 mg) were effective, though careful monitoring is required due to potential adverse effects such as glomerulonephritis, severe thrombocytopenia, and renal issues [78].

TTR stabilizers function by preventing the dissociation of TTR tetramers into monomers, a key step in the amyloid fibril formation process. A multicenter, international, double-blind, placebo-controlled, phase 3 trial randomly assigned 441 patients with ATTR in a 2:1:2 ratio to receive 80 mg of tafamidis, 20 mg of tafamidis, or placebo for 30 months [79]. In this study, tafamidis was associated with reductions in all-cause mortality and cardiovascular-related hospitalizations, and reduced the decline in functional capacity and quality of life as compared with placebo [79]. Importantly, the overall incidence and type of adverse events were similar in the tafamidis and placebo groups; the discontinuation of the trial drug owing to adverse events was less common in patients who received tafamidis than in those who received placebo, and dose reductions were uncommon and occurred more often in the placebo group [79].

Additionally, diflunisal, a nonsteroidal anti-inflammatory drug (NSAID), has demonstrated TTR-stabilizing effects and has been shown to slow the progression of familial amyloid polyneuropathy in small-scale studies [80,81]. One of the key advantages of diflunisal is its cost-effectiveness. However, it should be avoided in patients with severe renal dysfunction, advanced heart failure, or those at high risk of bleeding [82].

### 10.2. Therapies in AL

The treatment of AL primarily focuses on targeting plasma cell clones responsible for the production of amyloid fibrils, aiming to eradicate the pathological plasma cells and remove the affected light chain from the circulation. While the treatment of AL is predominantly driven by multiple myeloma therapies, the multisystem involvement in AL makes management more complicated and warrants the closer monitoring of drug side effects and toxicities [83]. Importantly, anti-plasma cell therapies do not have known cardiotoxicities and can thus be safely instituted in AL patients regardless of LVEF. While the treatment of AL is primarily managed by hematologists, it usually involves close collaboration with cardiologists to manage ongoing cardiac manifestations of the disease including congestive heart failure and arrhythmias.

For patients eligible for autologous stem cell transplantation (HDM/SCT), the standard of care has been high-dose melphalan, followed by HDM/SCT [84]. The eligibility of patients for HDM/SCT is based on the assessment of the risk of adverse outcomes, which is primarily determined by their cardiopulmonary status and baseline performance status. In experienced centers, treatment-related mortality has been reported to be ~3%, with long-term survival of up to 20 years being achieved in about 1/3 of patients treated with HDM/SCT [84].

Daratumumab, an anti-CD38 monoclonal antibody, in combination with cyclophosphamide, bortezomib, and dexamethasone (CyBorD or VCd) has emerged as the non-SCT therapy of choice in patients with newly diagnosed AL. In the ANDROMEDA study, this combination was associated with a remarkably high rate of deep hematologic responses with very good partial responses or better in ~79% of patients who received daratumumab plus CyBorD vs. 49.2% of patients who received CyBorD alone [85].

Treatment response in AL is closely monitored through measures such as serum and urine immunofixation electrophoresis, as well as free light chain levels, with a goal of achieving a complete or very good partial response [84]. In cases involving cardiac involvement, reductions in NT-proBNP levels are targeted to assess and improve cardiac function [85]. NT-proBNP is a biomarker that is directly modulated by amyloid light-chains and is universally accepted by AL specialists as a surrogate end point for survival [85].

### 10.3. Therapies in CS

The treatment of CS involves a combination of immunosuppressive therapies, arrhythmia management, and heart failure management, tailored to the severity and manifestations of the disease. Corticosteroids, primarily prednisone, are the cornerstone of therapy, aiming to reduce inflammation and granuloma formation in the heart [86]. A systematic review comprising 34 studies involving >1200 patients demonstrated that corticosteroids improve AV conduction in ~1/3 of patients and potentially prevent the worsening of systolic dysfunction, but the impact on survival is unclear [87]. Patients are often started on prednisone 0.5 mg/kg/day (generally 30–40 mg orally), with tapering guided by response [88].

In cases of refractory disease or where steroid-sparing is needed, other immunosuppressive agents such as methotrexate, azathioprine, or mycophenolate mofetil may be used as second-line agents. Methotrexate is usually administered in weekly doses of 10–20 mg, while the suggested dosing for azathioprine is 50–200 mg/day (dosed as 1–2 mg/kg body weight per day). Both methotrexate and azathioprine have a comparable side effect profile constituting leukopenia, hepatotoxicity, and gastrointestinal complications, although patients on azathioprine may be more susceptible to infection [89]. Biologic anti-tumor necrosis factor agents are next in line in the treatment of CS when other therapies have failed [90]. Prior to the initiation of treatment, the exclusion of latent tuberculosis and screening for hepatitis B, C, and HIV are mandated. Infection remains a significant concern with biological therapies.

For patients with significant arrhythmias, such as VT, antiarrhythmic drugs like amiodarone can be employed, and implantable cardioverter-defibrillators may be recommended for those at high risk of SCD [91]. In addition, standard heart failure treatments, including ACE inhibitors, beta-blockers, and diuretics, are used to manage symptoms and improve cardiac function in patients with heart failure. Monitoring the disease’s progression with imaging techniques like CMR and PET is essential for assessing treatment. As treatment can vary based on disease activity and organ involvement, a multidisciplinary approach, including cardiology, pulmonology, and immunology specialists, is crucial for optimal management.

## 11. Conclusions

In summary, granulomatous (specifically CS) and amyloidogenic cardiomyopathies both have overlapping clinical features, including heart failure and arrhythmias. Distinct differences in extracardiac “red flag” symptoms, echocardiographic features, and advanced imaging are key to distinguishing between the two conditions. In CA, echocardiography typically shows thickened heart walls, whereas in CS, there may be wall thinning with or without LV chamber dilation. CMR further differentiates the two, with CA often exhibiting global subendocardial and transmural LGE, while CS tends to show patchy, more localized areas of LGE. PET and Tc-PYP imaging further aid in diagnosis, with Tc-PYP uptake being a key marker for ATTR deposition in the absence of paraproteinemia and PET demonstrating active inflammation in CS. Disease-specific treatment for CS focuses on immunosuppressive therapies to reduce inflammation, while CA treatment involves stabilizing and/or silencing the TTR protein in ATTR and chemotherapy or stem cell transplant for AL. Timely diagnosis and appropriate management are crucial for improving outcomes in both conditions.

## Figures and Tables

**Table 1:** Extracardiac involvement of transthyretin cardiac amyloidosis, immunoglobulin light-chain amyloidosis, and cardiac sarcoidosis.

Transthyretin Cardiac Amyloidosis	Immunoglobulin Light-Chain Cardiac Amyloidosis	Cardiac Sarcoidosis
Carpal tunnel syndrome	Carpal tunnel syndrome	Mediastinal/hilar lymph nodes
Lumbar spinal stenosis	Lumbar spinal stenosis	Subcutaneous lymph nodes
Non-traumatic biceps tendon rupture	Non-traumatic biceps tendon rupture	Deep lymph nodes
Hip/knee replacement	Hip/knee replacement	Liver
Peripheral neuropathy	Peripheral neuropathy	Pulmonary
Orthostatic hypotension	Orthostatic hypotension	Spleen
Autonomic dysfunction	Autonomic dysfunction	Bone
	Gastroparesis	Muscle
	Macroglossia	Skin
	Nephrotic syndrome	
	Hepatic amyloidosis	

**Table 2:** Summary of key imaging-based studies contributing to diagnostic criteria of cardiac amyloidosis.

Study	Imaging Modality	Method	Key Finding
Phelan et al. [34]	Echocardiography	55 consecutive patients with CA were compared with 30 control patients with LV hypertrophy (HCM = 15, AS = 15).	CA is characterized by regional variations in LS from base to apex, and a relative ‘apical sparing’ patterm of LS accurately (sensitivity-93%. specificity-82%, AUC-0.94) differentiates CA from other causes
Pagourelias et al. [35]	Echocardiography	Retrospective study comprising 100 participants, including biopsy-proven CA (n = 40), HCM (n = 40) and hypertensive with myocardial remodeling	In patients with thickened hearts, ejection fraction longitudinal strain ratio has the best accuracy in detecting CA.
Martimez-Naharro et al. [52]	CMR	Prospective single-centered study comprising 263 patients with ATTR corroborated by grade 2 to 3 ^99m^Tc-DPD cardiac uptake, 17 with suspected ATTR (grade 1 ^99m^Tc-DPD), and 12 asymptomatic ATTRv; 50 patients with AL acted as disease comparators	ATTR patients demonstrate very elevated ECV values and typical subendocardial or transmural LGE pattern: ECV quantifies amyloid burden, and predicts adverse outcomes including death
Karamitsos et al. [50]	CMR	53 AL patients (cardiac involvement: none = 14, possible = 11, definite = 28).compared with 36 healthy participants and 17 individuals with AS. cardiac amyloid patients.	Noncontrast TI mapping has high diagnostic accuracy for detecting cardiac AL, and is potentially more sensitive for detecting early disease than LGE imaging
Fontana et al. [51]	CMR	CA subtypes comprising ATTR (n = 85). ATTRv (n = 8) and AL (n = 79) were compared with 52 healthy volunteers and 46 patients with HCM	Native myocardial Tl mapping detects ATTR with similar diagnostic performance and disease tracking to AL, but with lower maximal Tl elevation, and appears to be an early disease marker.
Perugini et al. [65]	Nuclear scintigraphy	25 biopsy-proven CA patients (ATTR-15, AL-10) and 10 controls underwent ^99m^Tc-DPD scintigraphy	^99m^Tc-DPD uptake was present in all ATTR patients and absent in all AL patients and controls, thereby demonstrating an accuracy of 100% in distinguishing ATTR from AL
Bokhari et al. [68]	Nuclear scintigraphy	45 participants (AL-12, ATTRut-15 and ATTRv-17) underwent ^99m^Tc-PYP scintigraphy, and cardiac retention was assessed with both a semiquantitative visual score, and by quantitative analysis by drawing a region of interest over the heart corrected for contralateral counts and calculating a heart-to-contralateral ratio.	Using a heart-to-contralateral ratio >1.5 consistent with intensely diffuse myocardial tracer retention had a 97% sensitivity and 100% specificity with area under the curve 0.992 (*p* < 0.0001) for identifying ATTR.
Gillmore et al. [64]	Nuclear scintigraphy	Bone scintigraphy from 1217 patients, comprising biopsy-proven CA (n = 857) and non-amyloid cardiomy opathies (n = 360) were evaluated.	Grade ≥2 myocardial radiotracer uptake on bone scintigraphy and the absence of a monoclonal protein in serum or urine had a specificity and positive predictive value for cardiac ATTR amyloidosis of 100% (positive predictive value confidence interval, 98.0–100).

CA = cardiac amyloidosis; LV = left ventricle; HCM = hypertrophic cardiomyopathy; ATTR = transthyretin amyloidosis; ATTRwt = wildtype transthyretin amyloidosis; ATTRv = variant transthyretin amyloidosis; AL = light chain amyloidosis; C.R = cardiac magnetic resonance imaging: ECV = extracellular volume: LGE = late gadolinium enhancement; 99mTc-DPD = Technetium-99m-labelled 3,3-diphosphono-1,2-propanodicarboxylic acid; 99mTc-PYP = Technetium-99m-labelled pyrophosphate.

**Table 3:** Societal Recommendations on Diagnostic criteria for Cardiac Sarcoidosis.

Heart Rhythm Society 2014 [72]	Japanese Circulation Society 2016 (Criteria for Systemic Disease) [73]	World Association of Sarcoidosis and Other Granulomatous Disorders 2014 [74]
**Definite CS:** -Histological confirmation is based on the presence of noncaseating granuloma in the myocardial tissue with no alternative cause identified **Probable CS:** -Based on clinical diagnosis from invasive and noninvasive studies -There is a histological diagnosis of extracardiac sarcoidosis, and I of the following is present: • Immunosuppressant-responsive cardiomyopathy or AVB • Unexplained systolic dysfunction (LVEF <40%) • Unexplained sustained VT or high-degree AVB • LGE on CMR in a pattern consistent with CS • Patchy FDG uptake on a dedicated cardiac PET in a pattern consistent with CS • Positive ^67^Ga uptake in a pattern consistent with CS • Other causes have been reasonably excluded.	**Histological diagnosis:** -EMB or surgical specimens demonstrate noncaseating granulomas Clinical diagnosis (without biopsy): - ≥2 of the 5 major criteria are satisfied OR 1 in 5 major and ≥2 minor criteria are satisfied: -Major criteria: • High-degree AVB or fatal VT/VF • Basal thinning of the ventricular septum or abnormal ventricular wall anatomy • LV contractile dysfunction • ^67^Ga or FDG-PET reveals abnormally high tracer uptake in the heart • CMR reveals LGE of the myocardium -Minor criteria: • Abnormal EKG • Perfusion defects on SPECT • Monocyte infiltration and moderate fibrosis on EMB AND • Granulomas are found in organs other than the heart OR the individuals show clinical findings strongly suggestive of pulmonary or ophthalmic sarcoidosis AND at least 2 of 5 characteristic findings of sarcoidosis are present: • Bilateral hilar lymphadenopathy • Elevated ACE • Elevated serum soluble interleukin-2 receptor levels • Significant tracer accumulation in Ga citrate scintigraphy or FDG-PET • A high percentage of lymphocytes in bronchoalveolar lavage fluid • with a CD4/CD8 ratio > 3.5	-Granulomatous inflammation has been demonstrated in another organ and I of the following: • Treatment-responsive cardiomyopathy and AVB • Reduced LVEF in the absence of other risk factors • Spontaneous or inducible sustained VT with no risk factors • High-degree AVB • Patchy uptake on a dedicated cardiac PET • LGE on CMR in a pattern suggestive of CS • Positive ^67^Ga uptake • Defect on perfusion scintigraphy or SPECT scan • T2 prolongation on CMR • Other causes have been reasonably excluded

CS = cardiac sarcoidosis; AVB = atrioventricular block; LGE = late gadolinium enhancement; LVEF = left ventricular ejection fraction; CMR = cardiac magnetic resonance imaging; FDG = Fluorine-18 fluorodeoxyglucose; PET = positron emission tomography; ACE = angiotensin converting enzyme; VT/VF = ventricular tachycardia/ventricular fibrillation; EMB = endomyocardial biopsy.

## Data Availability

Data sharing is not applicable.
